# Shape-preserving elastic solid models of macromolecules

**DOI:** 10.1371/journal.pcbi.1007855

**Published:** 2020-05-14

**Authors:** Guang Song

**Affiliations:** 1 Department of Computer Science, Iowa State University, Ames, Iowa, United States of America; 2 Program of Bioinformatics and Computational Biology, Iowa State University, Ames, Iowa, United States of America; Bogazici University, TURKEY

## Abstract

Mass-spring models have been a standard approach in molecular modeling for the last few decades, such as elastic network models (ENMs) that are widely used for normal mode analysis. In this work, we present a vastly different elastic solid model (ESM) of macromolecules that shares the same simplicity and efficiency as ENMs in producing the equilibrium dynamics and moreover, offers some significant new features that may greatly benefit the research community. ESM is different from ENM in that it treats macromolecules as elastic solids. Our particular version of ESM presented in this work, named *α*ESM, captures the shape of a given biomolecule most economically using *alpha shape*, a well-established technique from the computational geometry community. Consequently, it can produce most economical coarse-grained models while faithfully preserving the shape and thus makes normal mode computations and visualization of extremely large complexes more manageable. Secondly, as a solid model, ESM’s close link to finite element analysis renders it ideally suited for studying mechanical responses of macromolecules under external force. Lastly, we show that ESM can be applied also to structures without atomic coordinates such as those from cryo-electron microscopy. The complete MATLAB code of *α*ESM is provided.

## Author summary

Mass-spring models have been a standard approach in classical molecular modeling where atoms are modeled as spheres with a mass and their interactions modeled as springs. The models have been extremely successful. Thinking ahead, however, as molecular systems of our interest grow more quickly in size or dimension than what our computation resources can keep up with, some adjustments in methodology are timely. This work presents a vastly different elastic solid model (ESM) of macromolecules that shares the same simplicity and efficiency as mass-spring models in producing the equilibrium dynamics and moreover, offers some unique features that make it suitable for much larger systems. ESM is different from ENMs in that it treats macromolecules as elastic solids. Our particular version of ESM model presented in this work, named *α*ESM, captures the shape of a given biomolecule most economically using *alpha shape*, a well-established technique from the computational geometry community. Consequently, it can produce most economical coarse-grained models while faithfully preserving the shape. ESM can be applied also to structures without atomic coordinates such as those from cryo-electron microscopy.

## Introduction

Great strides have been made in the last few decades in determining the structure and dynamics of macromolecules. They are currently over 160,000 structures deposited in the PDB [1] and structures of increasingly larger molecular assemblies are becoming available. The cryo-electron microscopy, as one example, has brought much excitement to the field of structural biology, being able to not only determine at near-atomic accuracy the structures of extremely large complexes but also capture their dynamics through the determination of many conformations. Together with insights gained from computational modeling and simulations, the growing knowledge is transforming the field and will reveal much mechanistic understanding of many molecular systems.

Normal mode analysis in general and elastic network models in particular have been widely used in the last several decades for studying the equilibrium dynamics of macromolecules. Normal modes often reveal insightful clues about functional motions and high overlaps are often found when interpreting conformation changes using these modes. Elastic network models (ENMs) (see seminal work in Refs. [2–6]) are extremely simple and easy to use, and yet are able to accurately reproduce especially the low frequency normal modes. Other notable milestones in the development of ENMs include the RTB model [7] that extended the applications of ENMs to larger structure complexes [8], the non-linear block normal modes (NOLB) model by Grudinin and co-workers [9], the resonance-based BOSE model [10] by Na and Song and its application to HIV-1 capsid [11] that has nearly 5 million atoms, to name a few. A few other recent developments significantly increased the accessibility of ENMs and made them available to a much broader community, such as the ProDy python package [12], the iMod package for normal mode computations in internal coordinates [13], etc. For a recent review on elastic network or coarse-grained models of macromolecules, see Refs. [14, 15]. The success of ENMs was often attributed to its ability to capture the overall shape of macromolecules, which was recognized to have a dominant influence over their dynamics [8, 16, 17]. For symmetric complexes, shape, or symmetry to be more precisely, was shown to be the sole determinant of their motion patterns [18].

In this work, we present an elastic solid model (ESM) that is as simple and convenient-to-use as elastic network models such as ANM [5]. Moreover, it offers some additional significant features that may greatly benefit the research community.

ESM is different from ENM in that it treats macromolecules as solid blocks of certain shapes and material properties. The most distinct feature of the ESM presented in this work is that it captures the shape of a given macromolecule intentionally and most economically using the *alpha shape* [19], a well-established technique from the computational geometry community. We name our model *α*ESM (or alphaESM), short for ESM by alpha shape, to distinguish it from other ESM models constructed without using alpha shape [20–22]. By employing alpha shape, *α*ESM can produce most economical coarse-grained models while faithfully preserving the shape. After capturing the shape, instead of constructing a mass-spring model that consists of masses and springs, *α*ESM builds an elastic solid model composed of tetrahedra, which are conveniently available as the output of the alpha shape. Finite element method, a technique well established in engineering, is then applied to obtain the stiffness matrix, which is the counterpart of the Hessian matrix in ENMs. It follows that normal modes can be then computed from the stiffness and mass matrices. As a solid model that uses finite elements, ESM should be most suited for studying mechanical responses of biomolecular systems [20]. Lastly, we show that ESM is readily applicable also to structures without atomic coordinates such as those from cryo-electron microscopy.

Elastic solid models (ESMs) of macromolecules are not entirely new. An elegant elastic solid model was developed by Bathe [20] over a decade ago. Given a protein structure, the model first determined the boundary of a protein volume by computing the solvent-excluded surface using a software called MSMS [23]. The surface is then simplified using another software called QSLIM [24]. After obtaining the surface representation in the form of a triangle mesh, the model converts the triangle surface mesh into a 3-D volume mesh using the commercial program ADINA. The 3-D volume mesh, composed of tetrahedral elements, is then used to compute the stiffness and mass matrices. The drawbacks of Bathe’s model are several. First, it does not use coordinates of the given structure as nodes, and consequently, an extra step of interpolation from generated nodes back to atoms is required in order to obtain normal modes of the original atoms in the structure. Some nice properties of the modes such as orthonormality likely get lost in the process. Secondly, it uses external software such as ADINA that is not accessible to most readers, which reduces its usability. Third, the usage of multiple software makes streamlining the computation difficult. In contrast, *α*ESM uses the given atomic coordinates as nodes to represent a structure. Our entire program, which has only a few lines of code, is developed under MATLAB and is included for readers’ convenience. The program is extremely simple and ready to be deployed. For readers who do not have the MATLAB license, the included program may serve as a pseudo code, in that the detailed algorithmic steps described there should be readily translatable to other programming languages. Another elastic solid, instead of mass-spring, model of macromolecules that is closely related to our work was developed by Hinsen for computing waves in infinite protein crystals [21]. Our elastic solid model is designed for finite, individual macromolecules or complexes.

Another attractive feature of ESM is that it can be applied directly to electron microscopy (EM) density data. It is worth noting that ENM as well can be and had been applied to low resolution EM density data to compute dynamics and normal modes [25–28]. In order to do so, the EM density data was pre-processed using techniques such as vector quantization [29, 30]. Vector quantization produces a finite number of Voronoi cells whose centroids are optimally placed to best approximate the EM density. These centroids or code vectors, obtainable from software Situs [29], were then used as nodes in ENM to construct an elastic network and to compute normal modes. Besides code vectors [25–28], the EM density and volumetric data were approximated also with pseudo atoms [31, 32] or virtual particles [33]. All these models inevitably approximated the EM volume using a discrete set of points, which were then connected to form a mass-spring model for normal mode computations. Instead of approximating the density with a discrete set of points, *α*ESM creates an elastic solid representation of the entire density distribution through the application of alpha shape. It has a unified approach to structures with or without atomic coordinates.

## Methods

### The *α*ESM model

**Fig 1:**
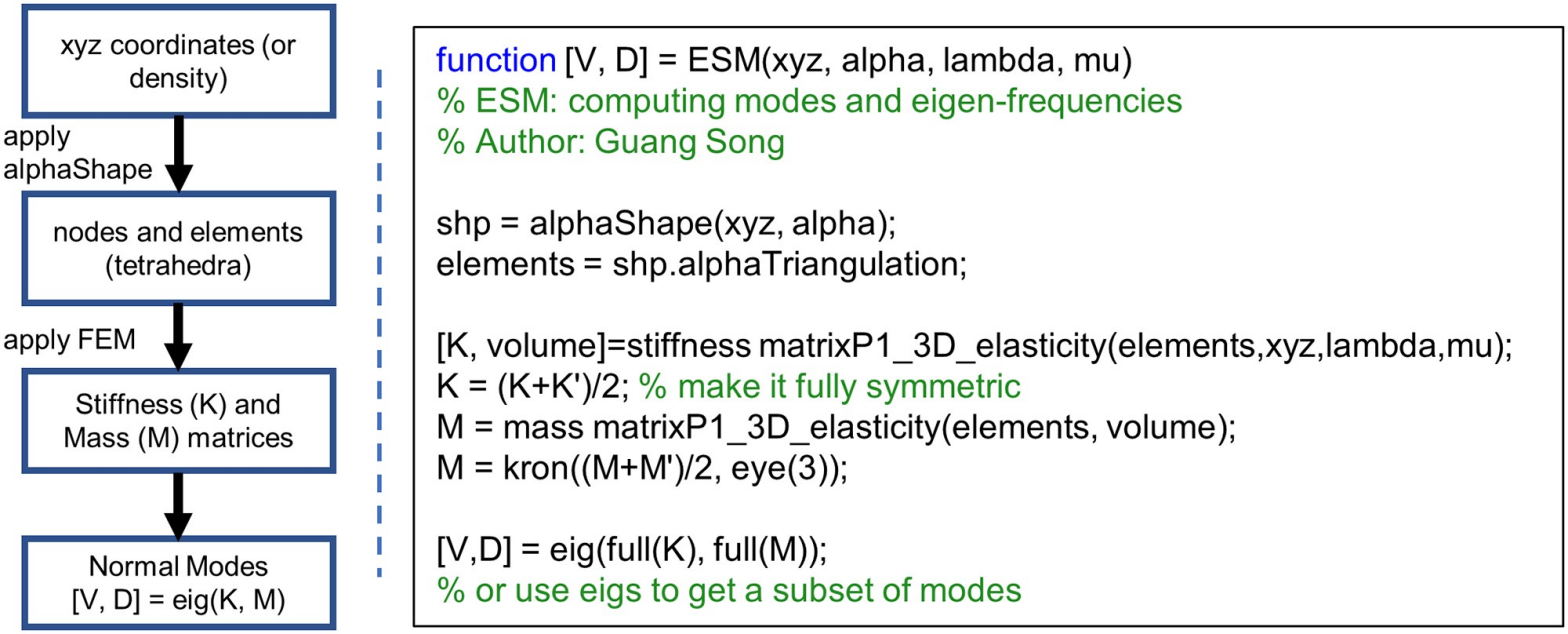
The flow chart of *α*ESM (left) and the corresponding MATLAB script (right). *alphaShape*, *kron*, and *eig* are built-in MATLAB functions. The two scripts for computing stiffness matrix and mass matrix are available at MATLAB file exchange (https://www.mathworks.com/matlabcentral/fileexchange/27826-fast-fem-assembly-nodal-elements), kindly contributed by Rahman and Valdman [34]. *xyz* are input coordinates, *alpha* is the only parameter of *alpha shape*, playing a role similar to the cutoff distance in ENM, lambda and mu are Lamé parameters (see text for more info). The above MATLAB script is available at S1 File.

Given a macromolecule structure, *α*ESM can be applied in a straight-forward manner as shown in Fig 1.

As shown in Fig 1, alpha shape [19] can be directly applied to the xyz coordinates (or density in a like manner). Intuitively, alpha shape works in the following way. Given a set of points in 3D, Delaunay triangulation [35] creates a tetrahedral mesh of all the points. Alpha shape applies a filter to the mesh and keeps only a subset of the tetrahedra that pass the filter. The filter that alpha shape uses is a sphere of radius alpha: only tetrahedra whose circumspheres’ radii are less than or equal to alpha are kept. For example, if an alpha of 10 Å is used, then the alpha shape will return a subset of tetrahedra (or quadruples of atoms) whose circumspheres’ radii are less than or equal to 10 Å. In this sense, the alpha value acts in a similar way to the cutoff distance in elastic network models. It is worth noting that the original alpha shape algorithm [19] actually keeps also edges and triangles (also called 1-simplex and 2-simplex respectively) that pass the filter, while the alpha shape implementation in MATLAB keeps only tetrahedra (also called 3-simplex). Alpha shape produces a volumetric representation of the structure using tetrahedral cells. This can be done in a single line of code in MATLAB (see Fig 1). The nodes of tetrahedra are the input coordinates. The tetrahedra themselves can be used as elements for any standard finite element analysis.

Once we have the nodes (say *C*^*α*^ atoms) and the elements (the tetrahedra), the stiffness matrix **K** and the mass matrix **M** can be obtained. The derivation of the stiffness and mass matrices can be found in most finite element method (FEM) textbooks [36] and will not be covered here. However, readers can readily use fast MATLAB scripts (kindly contributed by Rahman and Valdman [34]) to compute **K** and **M**, as shown in Fig 1, where λ and *μ* are Lamé parameters and can be computed from Young’s modulus *E* and Poisson ratio *ν* as follows:

$$\begin{array}{ccc} \lambda & = & \frac{\nu E}{\left(1+\nu )(1-2\nu \right)} \end{array}$$ (1)

$$\begin{array}{ccc} \mu & = & \frac{E}{2(1+\nu )} \end{array}$$ (2)

Intuitively, Young’s modulus (*E*) measures the stiffness of a solid material and is defined as the ratio of stress (or force per unit area) over strain (proportional deformation) under a uniaxial stretching or compression. Materials with a higher *E* are harder to stretch or compress than materials with a lower *E*, and thus appear stiffer. The **K** matrix is linearly proportional to *E*. In both its definition and its relationship to the stiffness matrix **K**, *E* is similar to spring constant *γ* used in elastic network models. *E* is an intrinsic material property of solid objects. For proteins, *E* is in the order of a few GPa, such as for actin, tubulin, or collagen [37], and is about 5.5 GPa for hydrated lysozyme crystals [38, 39] and 1.8 GPa for the capsid of bacteriophage *ϕ*29 [40]. An earlier work estimated that globular proteins should have a Young’s modulus in the range of 2 to 10 GPa [41]. In this work, *E* is a parameter that will be calibrated by fitting to the experimental B-factors, in a similar way to how the spring constant parameter is determined in some elastic network models [5]. The Poisson ratio is the negative of the ratio of traverse strain over axial strain and measures how much a material expands or shrinks horizontally when compressed or stretched vertically. The Poisson ratio for most material is in the range of 0.2 to 0.5. A Poisson ratio of 0.3 or 0.4 has been used for proteins [20, 40, 42, 43]. And it was shown that elastic behaviors of proteins depended only weakly on the exact value of Poisson ratio used [40]. In this work, a Poisson ratio of 0.3 is used.

It is worth noting that in FEM the mass matrix **M** is usually not diagonal, unlike the mass matrix used in ENMs. Additionally, the mass matrix **M** produced from the MATLAB script shown in Fig 1 is a *N* × *N* matrix, where *N* is the number of nodes. To match it with **K**, which is 3*N* × 3*N*, one can simply carry out a kronecker product:

$$\begin{array}{c} M=kron(M,I_{3}), \end{array}$$ (3)

where **I**_3_ is a 3 × 3 identity matrix.

The stiffness matrix **K** is similar to Hessian matrices in NMA or ENMs. To obtain normal modes, one can do:

$$\begin{array}{c} {}[V,D]=eig(K,M), \end{array}$$ (4)

where *eig* is the standard MATLAB routine for computing eigenvalues and eigenvectors (modes). The modes are stored in **V** in columns, while the eigenvalues are returned in **D** as a diagonal matrix.

### Overlap

To compute an overlap between a conformational change (or displacement) **d** and a give mode **v**_*i*_, one can compute their dot product:

$$\begin{array}{c} O_{i}=\frac{d\cdot v_{i}}{\left||d|\right|}. \end{array}$$ (5)

In case the mass matrix **M** is not an identity matrix, the modes are first mass-weighted:

$$\begin{array}{c} q_{i}=v_{i}M^{1/2} \end{array}$$ (6)

### Mean square fluctuations

B-factor is commonly thought to be related to mean square fluctuation <(Δ**R**_*i*_)^2^> as [5],

$$\begin{array}{c} B_{i}=\frac{8\pi^{2}}{3}<{\left(\Delta R_{i}\right)}^{2}>, \end{array}$$ (7)

though a large portion of it originates from static disorder in crystal lattice. Work by Kurinov and Harrison [44] and Hinsen [21] demonstrated that rather than thermal fluctuations, static disorder was the dominant contributor to B-factors at cryogenic temperature (about 100 K). At room temperature, it was estimated that thermal fluctuations contributed about equally as static disorder for Lysozyme [44]. Since the vast majority of crystal structures (about 95% [45]) were determined at low cryogenic temperature, great caution must to be taken when interpreting B-factors. It was suggested that MD-derived atomic fluctuations and cross-correlations [46] or NMA-derived atomic fluctuations [47] should be used instead.

Now since each mode’s potential energy amounts to $\frac{1}{2}k_{B}T$, the amplitude *A*_*i*_ of mode **v**_*i*_ can be determined from:

$$\begin{array}{c} \frac{1}{2}EA_{i}^{2}v_{i}^{T}Kv_{i}=\frac{1}{2}k_{B}T, \end{array}$$ (8)

where E is Young’s modulus, *k*_*B*_ is Boltzmann constant and T is temperature. Thus, we have,

$$\begin{array}{c} \frac{1}{2}EA_{i}^{2}\lambda_{i}=\frac{1}{2}k_{B}T, \end{array}$$ (9)

which leads to, $A_{i}^{2}=\frac{k_{B}T}{E\lambda_{i}}$.

Therefore,

$$\langle {\left(\Delta R_{i}\right)}^{2}\rangle =\frac{k_{B}T}{E}\sum_{k}\frac{{\text{v}}_{k,i}^{2}}{\lambda_{k}}=\frac{k_{B}T}{E}trace({\left[{\text{M}}^{-\frac{1}{2}}{\text{K}}_{m}^{-1}{\text{M}}^{-\frac{1}{2}}\right]}_{ii}),$$ (10)

where the subscript *ii* represents the i-th 3-by-3 diagonal block and **K**_*m*_ is the mass-weighted stiffness matrix, i.e., **K**_*m*_ = **M**^-1/2^**KM**^-1/2^. For ANM [5], it is:

$$\begin{array}{c} <{\left(\Delta R_{i}\right)}^{2}>=\frac{k_{B}T}{\gamma }trace\left({\left[H^{-1}\right]}_{ii}\right), \end{array}$$ (11)

where E is replaced by *γ* and **K** by the Hessian matrix **H**. Note that there is a slight difference between Eq (11) and that of GNM [3], which has an extra factor of 3 and **H** is replaced by Kirchhoff matrix **Γ** without applying the trace.

### What values to use for parameters alpha and Young’s modulus

Parameter alpha is very similar to the cutoff distance used in ENMs. If no alpha value is given, the alpha shape script of MATLAB will use the smallest alpha value that produces an alpha shape enclosing all the points. Based on our experience, an alpha value of 8 to 10 (in Å) is reasonable for coarse-grained *C*^*α*^-based models (for which ANM uses a cutoff distance of 13 Å [5]). The alpha value should be adjusted when a finer or coarser grained model is used. The adjustment can be done consistently by requiring the total volume of tetrahedral cells stays the same, while the number of cells may vary.

The Young’s modulus *E* on the other hand corresponds to the *γ* parameter used in GNM [3] and ANM [5]. For a given protein, like *γ*, *E* can be calibrated by scaling it so that the predicted B-factors match with the experimental B-factors. Again, this must be done with great caution since only a fraction of the experimental B-factors is from thermal fluctuations [21, 44].

### Vibrational spectrum

Given the eigenvalues λ_*i*_’s solved from the eigenvalue problem eig(**K**, **M**), the frequencies of normal modes *ω*_*i*_ can be computed as follows [48]:

$$\omega_{i}=\frac{1}{2\pi c}\sqrt{\lambda_{i}},$$ (12)

The conversion factor $\frac{1}{2\pi c}$ is used since it is customary to express *ω*_*i*_ in terms of the corresponding inverse wavelength of electromagnetic radiation (measured in cm^−1^). *c* is the speed of light and is 2.997925 × 10^10^ cm/sec. From *ω*_*i*_’s, the vibrational spectrum can be obtained by plotting its histogram.

## Results

In this section, we first present a comparison between ESM (*α*ESM in particular) and ENM (ANM [5] in particular). We then look at the mechanical response of macromolecules under external force as modeled by ESM and ENM respectively. Next, we show that ESM can be easily applied to extremely large structure complexes for normal mode computations and visualization. Lastly, ESM is applied to cryo-EM density maps.

### A comparison between ESM and ENM

**Fig 2:**
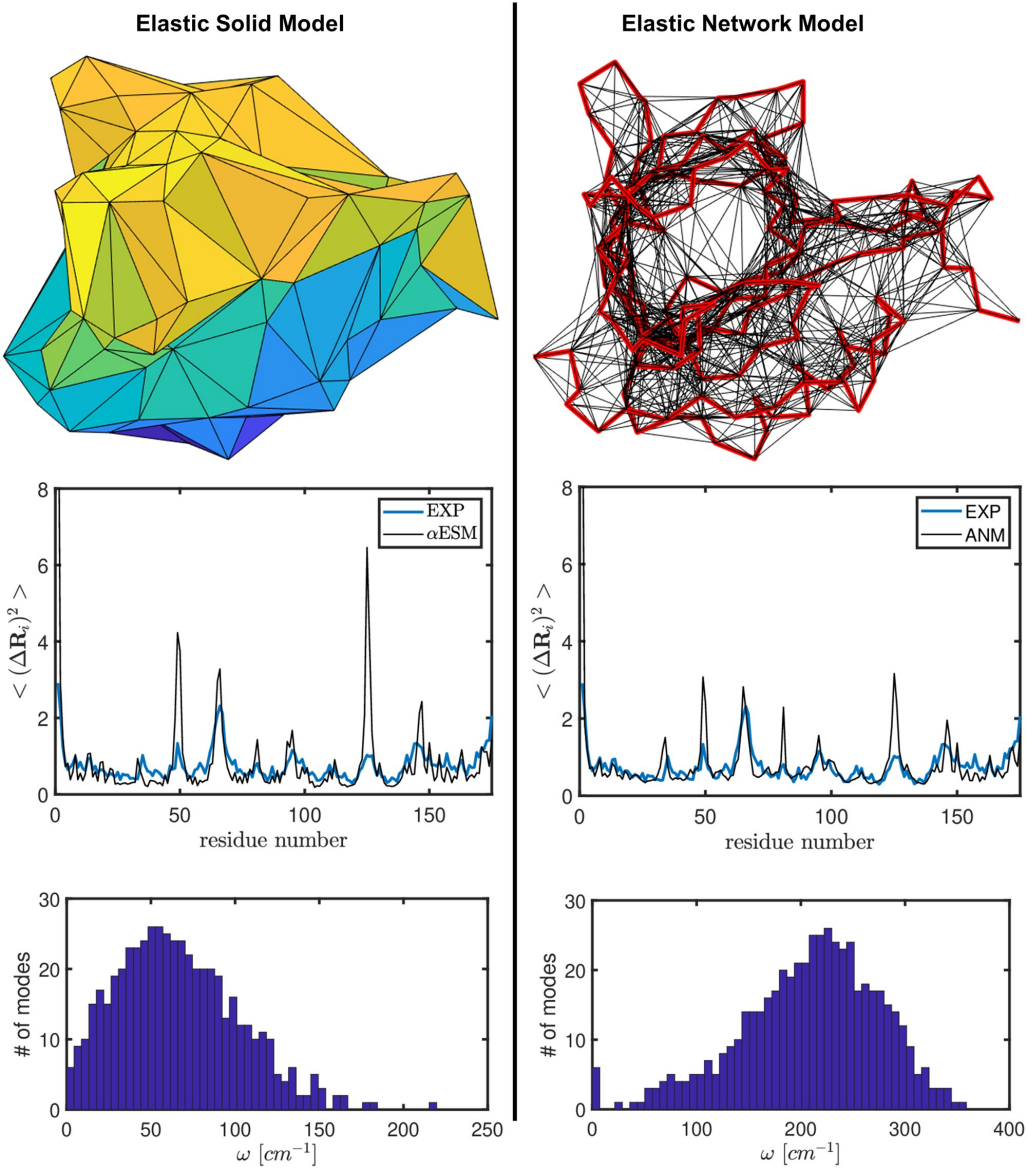
A contrast between ESM (here *α*ESM) and ENM (here ANM [5]), which model proteins differently as shown in the top row, followed by a comparison of their predictions on mean-square displacements (the middle row) and on vibrational spectra (the bottom row). The protein example shown is pig plasma retinol binding protein (183 residues, PDB-id: 1aqb) [49].

Fig 2 and Table 1 together present a detailed comparison between ESM and ENM, highlighting their similarities and differences.

**Table 1:** A comparison between ESM (here *α*ESM) and ENM (here ANM [5]) on model details.

	ESM (Elastic Solid Model)	ENM (Elastic Network Model)
Model input	coordinates of C^*α*^’s or all-atoms	the same
Representation	elastic solid	point masses connected by springs
Model unit	tetrahedron	point mass, spring
Interactions	continuous solid	mostly 2-body
Range of inter.	alpha	*r*_*cutoff*_
force constant	E = 0.47 Kcal/mol/Å^3^ (3.2 GPa)	*γ* = 0.39 Kcal/mol/Å^2^
lowest freq.	5.6 cm^−1^	40.8 cm^−1^
Normal modes	see S1 and S2 Videos	see S3 and S4 Videos
Conf. change	yes (see Table 2)	yes (see Table 2)
Convenience to use	easy	easy
Structure with xyz	yes	yes
EM density maps	directly	requires pre-processing
Deformation	more realistic	less realistic

There are a number of notable differences between the two models. First, ESM is continuous and volumetric and is composed of solid tetrahedra. ENM on the other hand is a mass-spring model (see Fig 2) and its interactions are usually just 2-body (see Table 1). The range of interactions is defined by a parameter called alpha (used by alpha shape) in our ESM model and a cutoff distance in ENM. As aforementioned, if no alpha value is given, the alpha shape script of MATLAB will use the smallest alpha value that produces an alpha shape enclosing all the points. Based on our experience, an alpha value of 8 to 10 (in Å) is reasonable for coarse-grained *C*^*α*^-based models (for which ANM uses a cutoff distance of 13 Å).

Both ESM and ENM can predict the B-factors fairly well (see the middle row comparison in Fig 2). Both have a correlation value of about 0.66/0.67 with experimental B-factors. Such comparisons with experimental B-factors allow one to calibrate the force constant: *γ* factor in ANM and the Young’s modulus *E* in ESM. In the particular example given in Table 1, fitting with experimental B-factors (assuming 100% contribution from thermal fluctuations) shows that the Young’s modulus is about 0.47 Kcal/mol/Å^3^, or 3.2 GPa, which is reasonable for globular proteins (the Young’s moduli of actin, tubulin, or collagen [37] and hydrated lysozyme crystals [38, 39] are similar). The value may be higher or even doubled since the actual contribution of thermal fluctuations in the B-factors of this structure (pdb-id: 1aqb), determined at room temperature, is probably less than 100%. To put Young’s modulus of this protein into perspective, such a material is comparable to nylon, whose Young’s modulus is 2 to 4 GPa, but softer than wood, whose Young’s modulus is about 10 GPa (https://en.wikipedia.org/wiki/Young%27s_modulus).

Given the force constant, the vibrational spectrum can be obtained (see the bottom row comparison in Fig 2). Both models have one dominant peak. For ESM, the first vibrational mode has a frequency of 5.6 cm^−1^, and for ENM, it is as high as 40.8 cm^−1^. Interestingly, the spectrum of ESM model (Fig 2) peaks at around 50-60 cm^−1^, matching fairly well in location with the first peak observed in experiments [50] that is at around 80 cm^−1^. The location of the second peak in the universal vibrational spectrum of all globular proteins [48] is at around 300 cm^−1^. This second peak is completely missing in ESM and might correspond to the dominant peak in ENM, in which the first peak is hardly present. Since here we have the spectrum result of only one protein, it is hard to draw any solid conclusion. It would be interesting to do a spectrum study on a large set of proteins of various sizes and/or classifications using these two models. Since this is not the main focus here, such a study is left for future work. However, the spectrum comparison does suggest that perhaps ESM may be better suited than ENM for computing the low frequency normal modes. Future studies will validate or invalidate this hypothesis.

**Fig 3:**
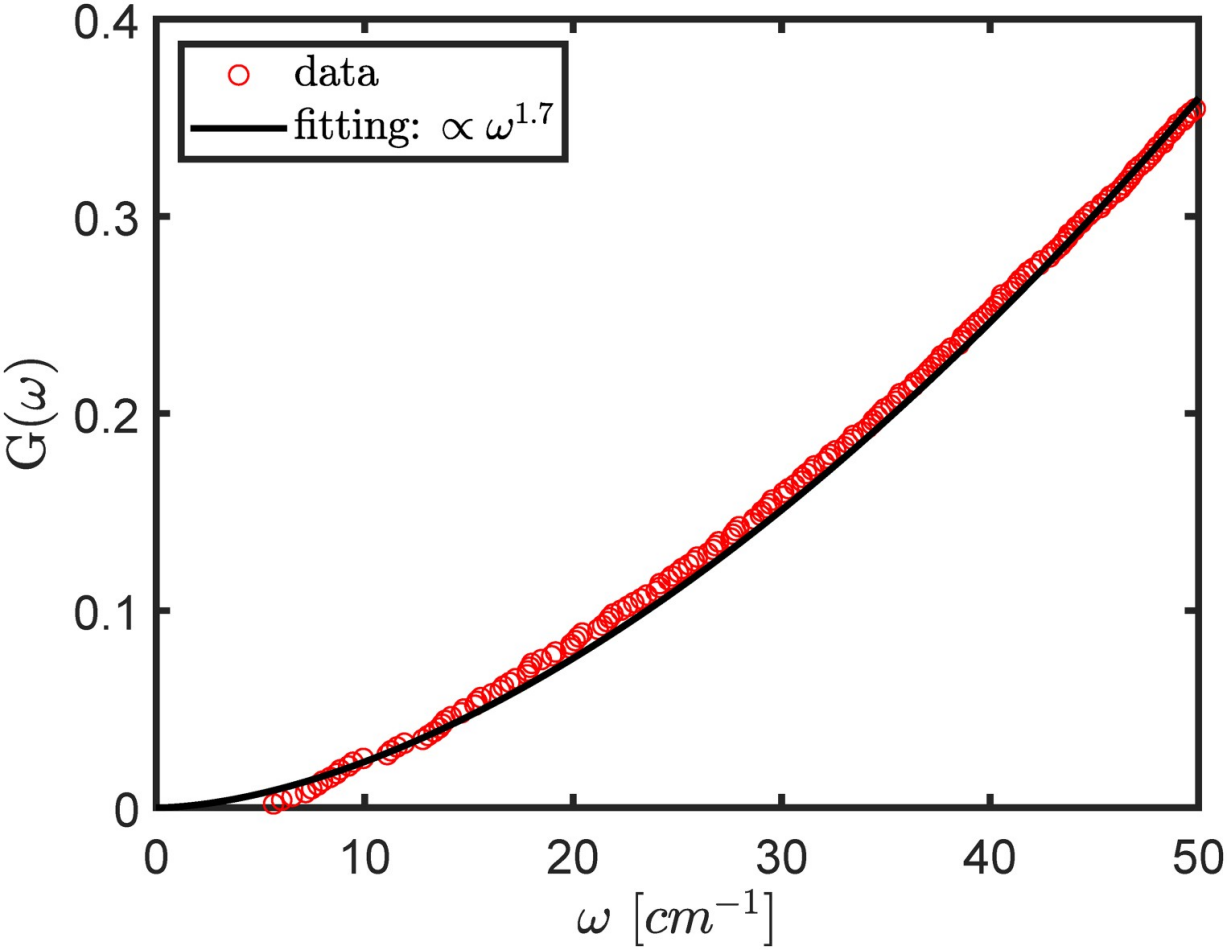
The fraction of modes up to frequency *ω*, *G*(*ω*), as a function of *ω*, as computed by ESM. The data is fitted with a power function with an exponent of 1.7.

It was noted that density of modes, or *g*(*ω*) (where *ω* denotes frequency), of globular proteins near the low frequency end increased linearly with the frequency [2, 51]. ben-Avraham [51] observed that the integration of *g*(*ω*), denoted as *G*(*ω*), or the fraction of modes below frequency *ω*, was related to *ω* by a simple power law, i.e., *G*(*ω*) ∝ *ω*^2^, from which it follows that, *g*(*ω*) ∝ *ω*. It is thus interesting to check if the normal modes of ESM proposed here also follow a simple power law. To this end, we plot *G*(*ω*) of ESM vs. *ω* in Fig 3. We fit the data with a power function and find that the best exponent is about 1.7, which is similar to what ben-Avraham and Tirion found [2, 51].

The normal modes of the two models are included in SI (see S1, S2, S3 and S4 Videos). The two sets of modes share significant similarities. Their differences also are apparent, probably since one is a mass-spring model and the other is an elastic solid model.

Another major application of normal mode analysis using either ESM or ENM is to interpret conformational changes. Apparently, both models can be easily applied to compute overlaps between a given conformation transition, say from an open form to a closed form, and the normal modes. In Table 2 we compare *α*ESM and ANM [5] with one of the state-of-art models iMOD [13] regarding their performance in interpreting conformational changes. To this end, we use the same benchmark dataset of 23 pairs of proteins as used by iMOD [13] and the same metrics such as *α*_1_, *α*_2_, *α*_3_: the top three best overlap values, *δ*_3_, *δ*_5_, *δ*_10_: cumulative overlaps of the top 3, 5, 10 modes, *Nα*_1_: the index of the mode that gives the best overlap, and *Nσ*_90_, the number of modes required to cover 90% of the modal variance [13]. From the table it is seen that the three models are mostly on par to one another. One noticeable difference, however, is that *α*ESM requires significantly fewer modes to cover 90% of the modal variance (low *Nσ*_90_). This perhaps correlates with the fact that the first peak of the vibrational spectrum of *α*ESM is reproduced more accurately (Fig 2). In all these computations, ANM uses a cutoff distance of 13 Å. *α*ESM uses an alpha value of 8 Å, except for one protein (1bnc.pdb) a slightly larger value (8.43 Å) is used in order to form a single connected shape. Results of individual protein pairs are included in S1 and S2 Tables.

**Table 2:** A comparison between *α*ESM, ANM [5], and iMOD [13] in interpreting conformational changes using the same benchmark protein dataset and metrics as used in the iMOD work by Chacon and coworkers [13].

	*α*_1_	*α*_2_	*α*_3_	*δ*_3_	*δ*_5_	*δ*_10_	*Nα*_1_	*Nσ*_90%_
Open to closed								
iMod	0.77	0.30	0.23	0.86	0.89	0.92	1.3	90
ANM	0.76	0.37	0.22	0.84	0.89	0.92	1.6	69
*α*ESM	0.75	0.37	0.24	0.85	0.91	0.94	1.3	**28**
Closed to open								
iMod	0.63	0.38	0.28	0.71	0.80	0.86	2.2	125
ANM	0.61	0.37	0.28	0.68	0.76	0.86	2.5	184
*α*ESM	0.64	0.37	0.30	0.74	0.82	0.89	2.0	**54**

All results represent average values over the 23 pairs of proteins in the dataset. Results of individual protein pairs can be found in S1 and S2 Tables. The three models are mostly on par to one another. One notable difference, however, is that *α*ESM requires significantly fewer modes (low *Nσ*_90_, highlighted in bold) to cover 90% of the modal variance. *α*_1_, *α*_2_, *α*_3_: top three best overlap values, *δ*_3_, *δ*_5_, *δ*_10_: cumulative overlaps of top 3, 5, 10 modes, *Nα*_1_: the index of the mode that gives the best overlap, and *Nσ*_90_, the number of modes required to cover 90% of the modal variance [13].

Up to this point ESM and ENM are fairly comparable. By trying them out, one should be able to easily find out that both models are extremely easy to use and can be applied directly to structures with coordinates, say those from the Protein Data Bank.

So why ESM? Besides offering an interesting alternative to ENM by employing a solid model, ESM does offer some additional features that researchers in the community may find attractive. First, since ESM is solid-based and uses tetrahedra as basic units or elements, a well-established technique widely used in engineering called finite element method (FEM) can be readily applied. FEM is a mature technique for studying mechanical responses of systems due to external forces. By adopting an elastic solid model of macromolecules, techniques developed in the field of FEM can be transplanted here to study the mechanical responses of macromolecules [20]. Secondly, as aforementioned, ESM can capture the shape of a given macromolecule intentionally and economically. Moreover, mature techniques exist in the field of computational geometry and computer graphics for simplifying a representation while preserving the shape. As a result, ESM can accurately represent large structure assemblies using much fewer variables/nodes than ENM. Since the computational efficiency of both ESM and ENM involves mostly solving an eigenvalue problem of a matrix whose cost is proportional to the number of variables/nodes used, the fewer-nodes-required solid representation of ESM should be more efficient than ENM, especially for very large complexes. Lastly, the cryo-EM data are becoming prevalent. Other low resolution data also are becoming increasingly more available. Another desirable feature of ESM is that it can extract a shape out of the low resolution density data. The rest of ESM can then be applied to obtain their normal mode dynamics.

In the remaining sections, we will attempt to illustrate these advantages.

### Deformation under external forces

Finite element method (FEM) is a mature, well-established technique for studying the mechanical response of systems under external force. It has been successfully applied to produce highly realistic simulations of processes such as bending, buckling, indentation, etc. over countless types of materials, including even the deformations of a viral capsid model [43]. Consequently, elastic solid models are expected to be better suited for reproducing the mechanical response of proteins and other biomolecules than elastic network models [20]. However, to the best of our knowledge, no direct comparison between elastic solid models and mass-spring models regarding structural deformation under external forces has been done before.

**Fig 4:**
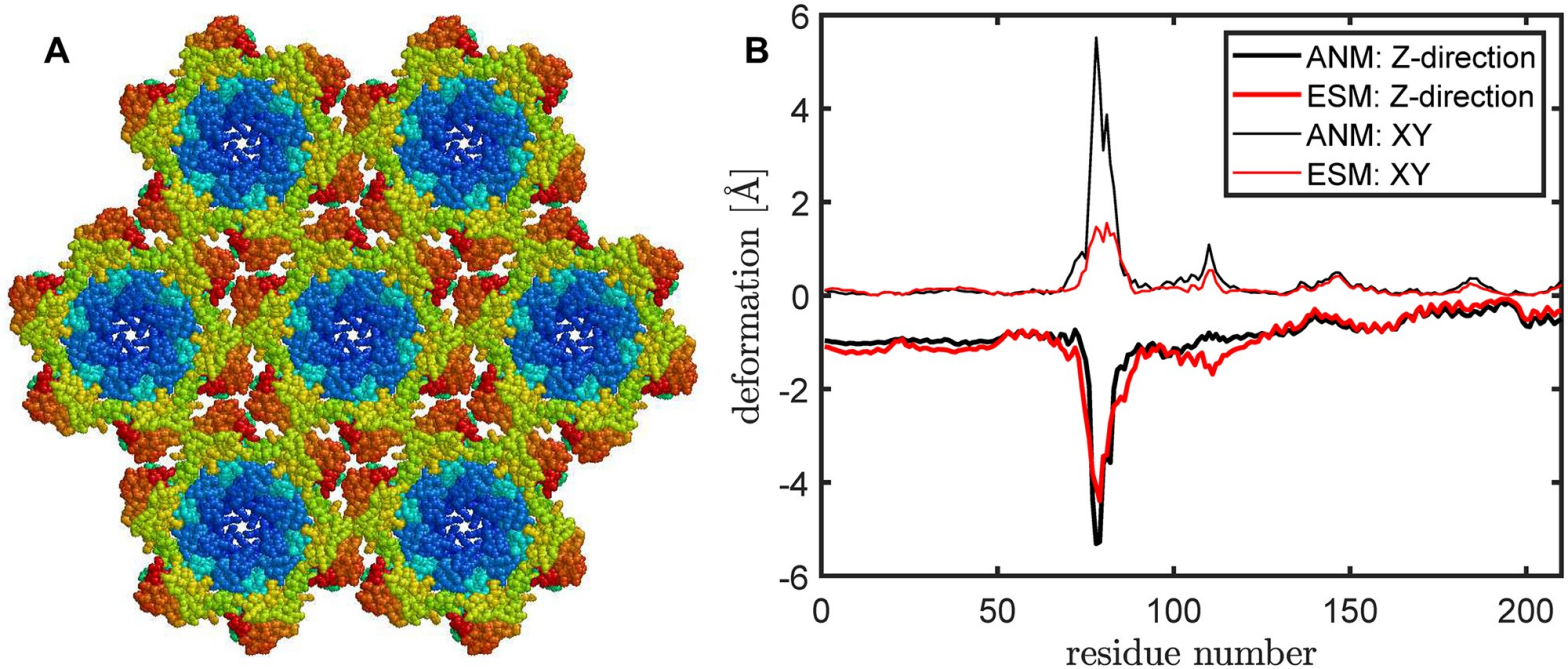
The structural deformation of a hexa-hexamer under external force. (A) A hexa-hexamer assembly of the HIV-1 capsid proteins (pdb-id: 4xfx) [53], obtained by applying the crystallographic symmetry. (B) The extents of deformation along Z (thick lines) or XY direction (thin lines) of the central hexamer under external force as computed by ANM (in black) and ESM (in red).

HIV-1 capsid proteins form closed conic structures or tubular capsid assembly [52]. Fig 4A shows a hexa-hexamer assembly of HIV-1 capsid proteins (pdb-id: 4xfx) [53], obtained by applying the crystallographic symmetry. Each hexamer is compose of six capsid protein chains of 231 residues long. For the particular capsid protein considered here (PDB-id: 4xfx [53]), only 216 residues are present in the solved crystal structure, out of which 6 residues on the N-terminal hairpin are further excluded since they are disconnected from the rest of the protein chain. As a result, each hexamer has 210*6 = 1260 residues.

The mechanical response of the HIV-1 capsid assembly under external force had been studied experimentally using atomic force microscopy (AFM) [54]. In our computational setup, we have only a hexa-hexamer (Fig 4A) and moreover, only the central hexamer is free to move, with all the surrounding hexamers fixed in space. We choose this much simplified setup since our purpose here is to compare between ENM and ESM, to see how different their mechanical responses are.

The extents of deformation are shown in Fig 4B. The ESM results are in red and ENM [5] in black. A constant force is exerted along the negative-Z direction on the *C*^*α*^’s of residues 83-91 and 115-117 since these residues are on the top. Fig 4B shows that, for ESM, the deformation has two peaks and is mostly along Z-axis (with a much smaller displacement horizontally) as one would expect. For ENM, the extent of deformation along the horizontal plane is as big as that along the vertical axis. The whole deformation process is captured in movies (see S5 and S6 Videos). Though the results here from one single example are not conclusive, it does suggest that the less realistic deformation by ENM may be attributed to its intrinsic mass-spring model, which is not best suited for simulating the deformation of materials. On the other hand, ESM captures the material property of the biomolecule and its deformation more accurately.

It is worth noting that mechanical responses computed through ESM and ENM as shown above or by other authors [55, 56] are limited since they are linear responses and theoretically, are valid only for infinitesimal displacements and forces. As an elastic solid model, ESM has another significant advantage over ENM: its tight connection with finite element analysis (*FEA*) allows commercial *FEA* software such to abaqus (Dassault Systèmes, USA) to be readily applied to produce highly accurate and realistic mechanical responses over a broad range of structural deformations. This is demonstrated in the following example.

### A tight connection between ESM and finite element analysis (FEA)

One significant advantage of a solid model of proteins over a mass-spring model is its tight connection to finite element analysis. The solid model of macromolecules produced by ESM naturally partitions a whole macromolecular structure into tetrahedra cells, which can be conveniently used as finite elements in finite element analysis. The tight connection between ESM and *FEA* allows commercial *FEA* software such to abaqus (Dassault Systèmes, USA) to be readily applied to produce highly accurate and realistic structure deformations and mechanical responses.

**Fig 5:**
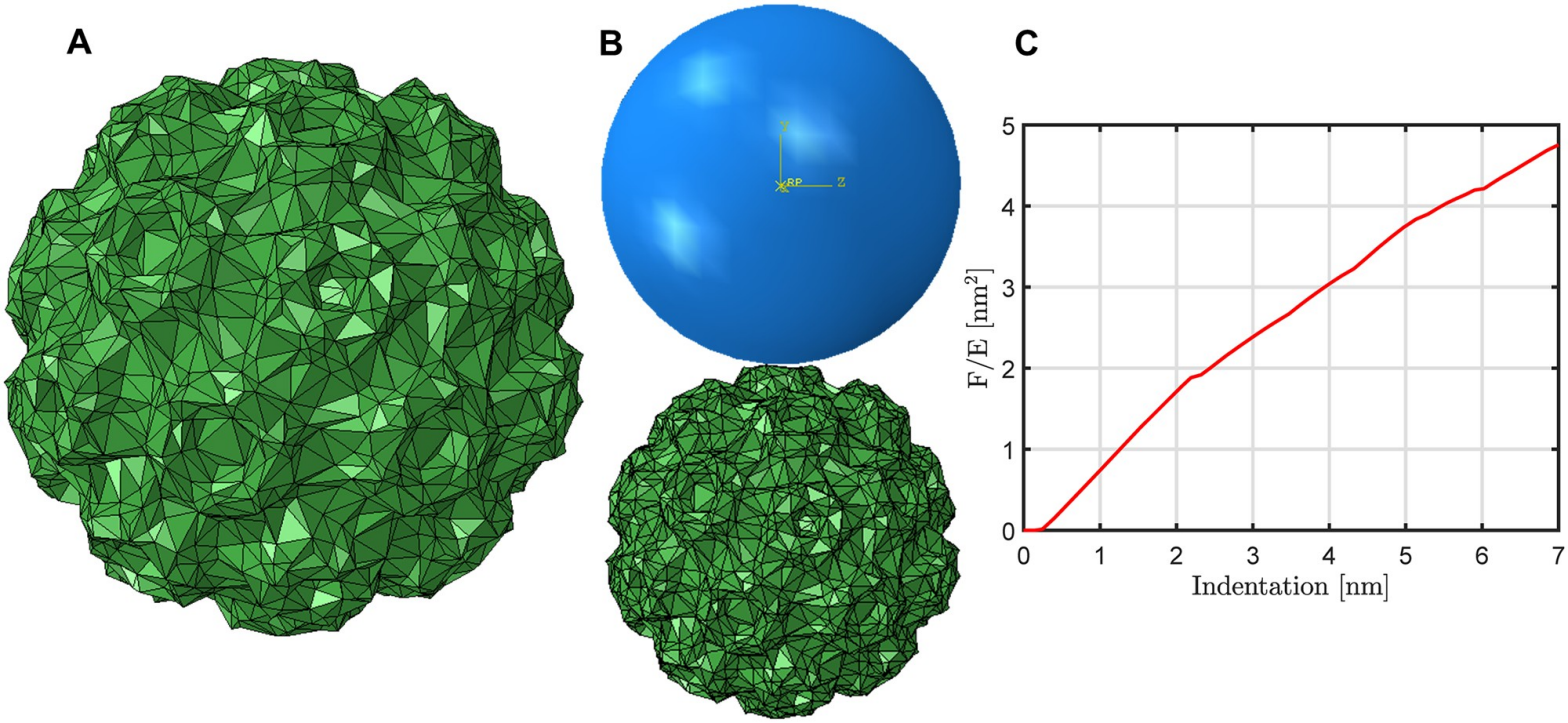
The mechanical response of a virus capsid under indentation. (A) A solid model of CCMV (cowpea chlorotic mottle virus) capsid (pdb-id: 1cwp) [57]. (B) The indentation setup: the spherical tip of AFM, which is modeled as a sphere (in blue), is placed on top of the capsid. (C) A plot of the vertical response force as a function of the indentation distance.

Michel et al. [43] applied *FEA* to study the mechanical response and stiffness of the capsid of cowpea chlorotic mottle virus (or CCMV, pdb-id: 1cwp [57]). The computed stiffness was then compared with experimental results obtained from an atomic force microscope (AFM) [43]. However, in Michel et al. [43]’s computation, the virus capsid was roughly approximated with a uniform spherical shell of a given radius and thickness. In the following, ESM is applied to CCMV capsid first to generate a tetrahedral volume mesh model (see Fig 5A). The model is built with every third *C*^*α*^ of the protein chains in the capsid and an alpha value of 12.91 Å. This particular alpha value is used so that the resulting model has the same volume as the model built with every *C*^*α*^ atom and an alpha value of 10 Å, a recommended alpha value for *C*^*α*^-based models. The model (Fig 5A) has 9,540 nodes and 52,296 tetrahedral elements. The mesh model is then fed into a FEA program abaqus. In addition to the capsid, the AFM spherical tip is modeled as a rigid sphere of 14 nm as in Ref. [43]. The indentation process is modeled by pressing the spherical tip downward onto the capsid (see Fig 5B) (with the bottom of the capsid held fixed in space). The mechanical response of the capsid is captured in the movie file S7 Video. Specifically, the correlation between the vertical response force and the indentation distance is plotted out in Fig 5C, from the slope of which the stiffness of the capsid per unit of *E*, or *κ*_*cal*_ can be computed. Note that the stiffness of the capsid is linearly proportional to the capsid’s Young’s modulus *E*. Next, by comparing *κ*_*cal*_ with the stiffness of capsid measured by AFM (*k*_*exp*_), which is 0.15 nN/nm [43], we obtain the Young’s modulus of the capsid. The slope *κ*_*cal*_ is 0.65 *nm* per unit of *E* (which is *nN*/*nm*^2^ or *GPa*). To find out the Young’s modulus *E* of the capsid, we write,

$$\begin{array}{c} E\kappa_{cal}=k_{exp}, \end{array}$$ (13)

from which we have,

$$\begin{array}{c} E=\frac{k_{exp}}{\kappa_{cal}}=\frac{0.15}{0.65}=0.23\;GPa=230\;MPa. \end{array}$$ (14)

This number is comparable to the 140 MPa predicted by Michel et al. [43]. Michel et al.’s predicted value is lower than ours probably due to the fact that they modeled the capsid as a perfect spherical shell with uniform thickness, while in reality, the capsid surface is not smooth at all but has significant ups and downs (see Fig 5B). To compensate this, the Young’s modulus predicted from our model has to be higher in order to have the same appearing stiffness.

The Young’s modulus thus calibrated by experimental AFM measurement can in turn be used in ESM to reproduce the normal modes of the capsid, specifically the magnitudes.

It is worth noting that the Young’s modulus predicted by our *α*ESM model depends on the alpha value used. Further studies are needed to remove the somewhat arbitrariness in the choice of alpha value in *α*ESM.

### Non-uniform coarse-graining and applications to extremely large complexes

The most common coarse-graining used in ENM is from all-atom to *C*^*α*^ only. If further coarse-graining is needed, uniform coarse-graining is usually applied, such as every 10th, 20th, or 40th *C*^*α*^’s [58]. Uniform coarse-graining does not intentionally preserve shape and may lose some important structure features.

An advantage of the solid representation of structures employed in ESM is that it allows convenient, non-uniform coarse-graining while intentionally attempting to preserve the overall shape.

In our ESM, a structure is represented by its alpha shape, a solid model of the structure. The surface of the model, which is represented by a surface mesh of triangles, is conveniently available from the resulting alpha shape. To coarse-grain a solid model, one can first simplify its surface mesh. A number of algorithms/tools exist for such a task, such as QSLIM [24], triangulated surface mesh simplification (https://doc.cgal.org/latest/Surface_mesh_simplification/index.html) [59, 60] in CGAL [61], or edge-collapse decimation on manifold meshes using libigl [62], etc. In our work, the MATLAB built-in function *reducepatch* is used. *reducepatch* reduces the number of surface triangles down to a user-specified percentage while attempting to preserve the shape of the original object.

**Fig 6:**
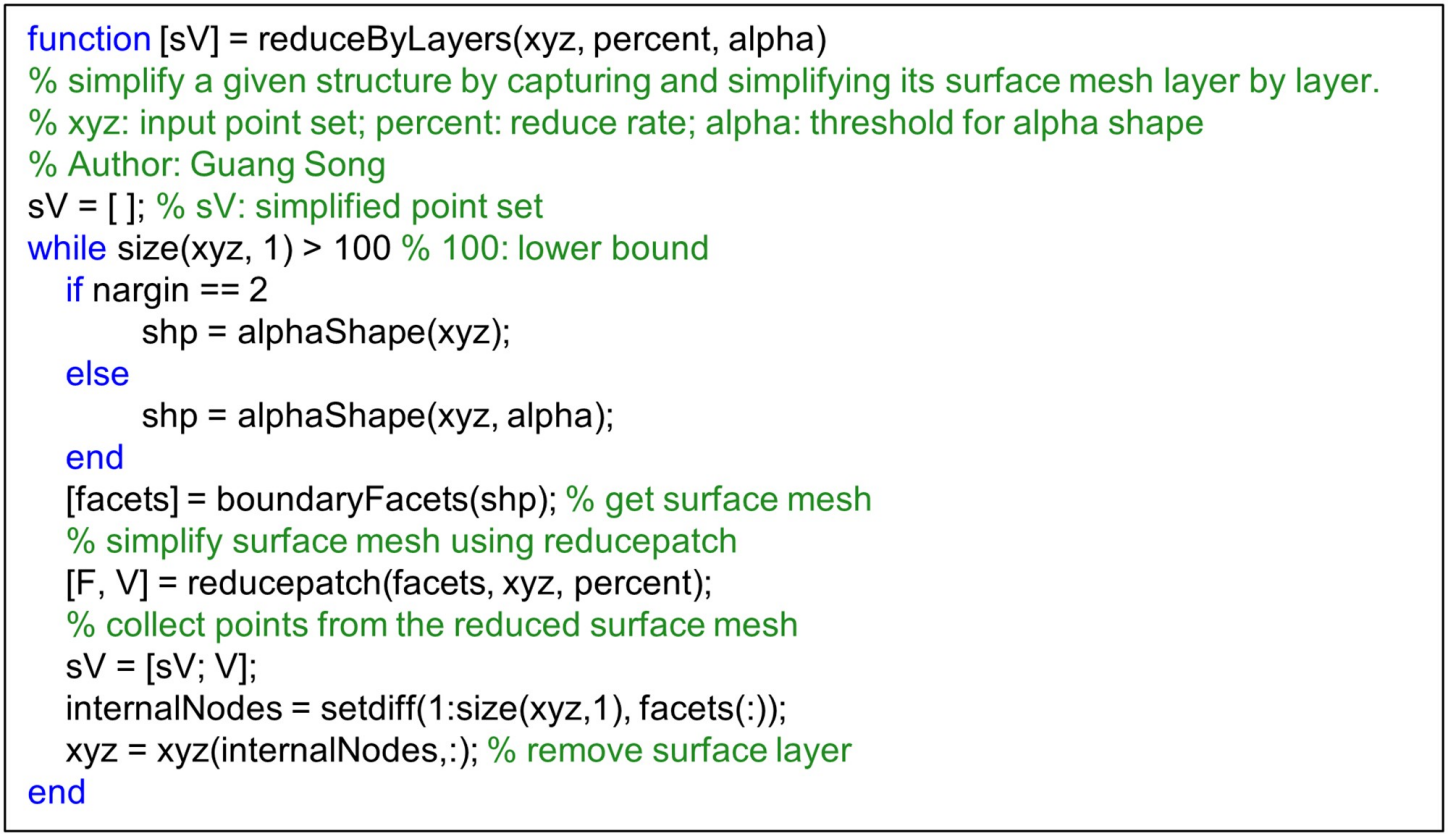
A MATLAB script for coarse-graining a structure represented by a point set by simplifying its surface mesh layer by layer. The script is available at S2 File.

The next obvious question is: how to reconstruct a new tetrahedral volume mesh after the surface mesh is simplified? What to do with the internal nodes below the surface? This turns out to be a non-trivial task, since a volumetric reconstruction may not always maintain the surface mesh. There are several alternatives. One is to discard the internal nodes altogether (i.e., keep only the surface mesh) and apply tools such as TetGen [63] or GMSH [64] to convert the surface mesh into a tetrahedral volume mesh. These programs however either create sharp long skinny tetrahedra or have to regenerate some internal nodes. Instead of regenerating internal nodes from scratch, another option is to use the existing internal nodes but reduce them to the same percentage as surface nodes. This can be done by applying a 3-D point cloud down sample algorithm [65], such as the *pcdownsample* function [65] available in MATLAB. In this work, we use the scheme given in Fig 6 to coarse-grain a given structure layer by layer while preserving its shape. The coarse-grained structure is then fed into ESM (Fig 1) to construct a volume mesh and to compute normal modes and eigen-frequencies. Note that internal nodes are reduced to the same percentage as surface nodes in the scheme given in Fig 6. This is intentionally done so that re-applying alphaShape using the ESM script (Fig 1) will produce a proper surface. If a different approach is employed to reconstruct the volume mesh, the internal representation of the structure can be coarser than that of the surface and fewer internal nodes may be used, as they matter only for numerical precision.

**Fig 7:**
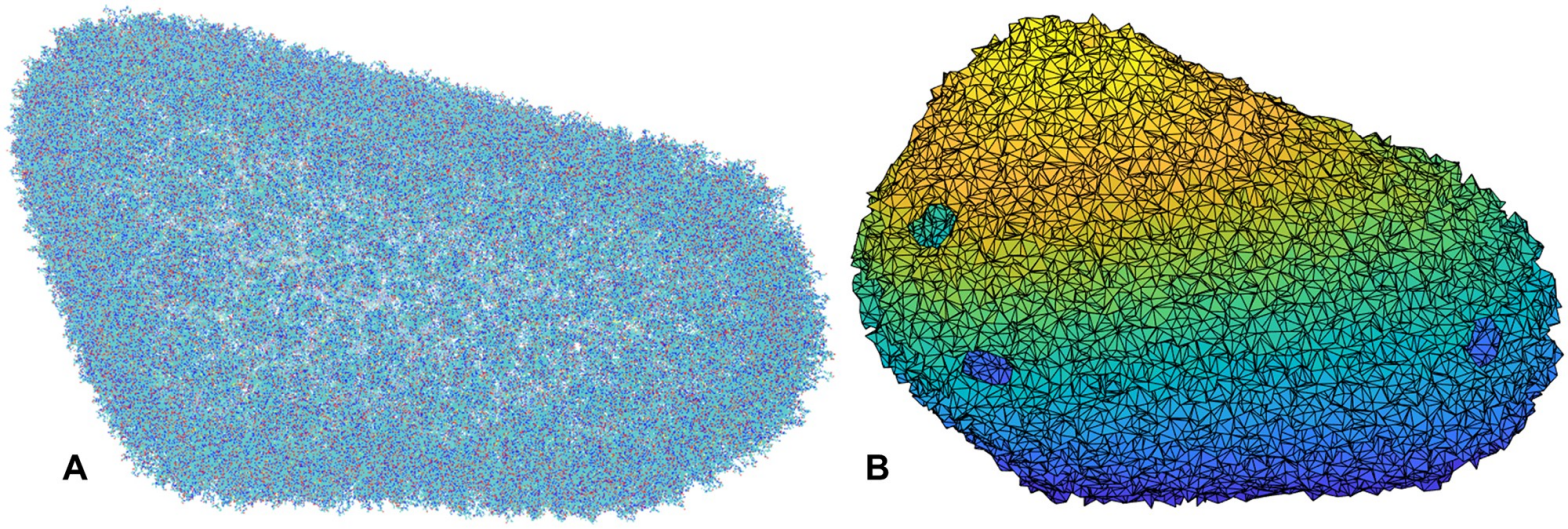
Structural models of HIV-1 capsid. (A) in all-atom representation, with over 2 million heavy atoms, and (B) in a simplified solid model, which has 13,747 nodes and consists of 67,023 tetrahedra. Even using *C*^*α*^’s only, the capsid would still have nearly 300,000 atoms.

Fig 7 shows the all-atom representation of HIV-1 capsid (PDB-id: 3j3q [52]). Because of the vast size of the structure, with over 2 millions heavy atoms present, the details are overwhelmingly too many and too small. Fig 7B shows the same structure in a simplified solid model and as a result, visualization of the structure becomes much more manageable (the holes on the surface are the locations of the pentamers, which are intentionally left out).

Using this solid model (about 13k nodes and 67k tetrahedra), not only the shape of the whole capsid is accurately preserved, the normal modes of the whole capsid can be easily obtained using the rest of ESM. A number of these modes (modes 7 and 8, the first two non-trivial modes, as well as mode 50) are included in S8, S9 and S10 Videos. The first few modes show breathing motions of the capsid, which appears to some extent like the breathing of a fish!

### Application to EM models

Another attractive feature of ESM is that it can be readily applied to study the dynamics of structures represented by cryo-EM density data. EM map files are available from the EM databank [66] and contain density values on a 3-D grid. For those who do not want to write their own program, one convenient way to extract the density information is to use program Situs [29], which has a *vol2pdb* command that extracts the density information (as well the corresponding coordinates) into a PDB format like file that contains a list of points in space and associated densities. In the process of running vol2pdb, a density threshold is required, and only densities above the threshold are exported.

Now to obtain dynamics from the cryo-EM density map, one needs a way to first represent the structure and its shape. To use all the points from the EM map is cumbersome and for some cases, infeasible. An established approach that uses a limited number of points to approximate the density distribution is called vector quantization [29, 30]. When applied to EM density, vector quantization produces a finite number of Voronoi cells whose centroids are placed to best approximate the EM density. These centroids, also called code vectors, were then used in constructing coarse-grained representations of structures [25]. The quality of these representations, however, depends on the number of code vectors used. One potential drawback of this approach is that it is not obvious how many code vectors are needed to represent the overall shape of a given structure: the number of code vectors needed may be rather arbitrary and depends on the particular structure under consideration and the user’s visual assessment, which often tends to be subjective. Another drawback is that vector quantization was not designed to preserve shape, but to best approximate the EM density distribution. The approach by nature employs a uniform coarse-graining.

**Fig 8:**
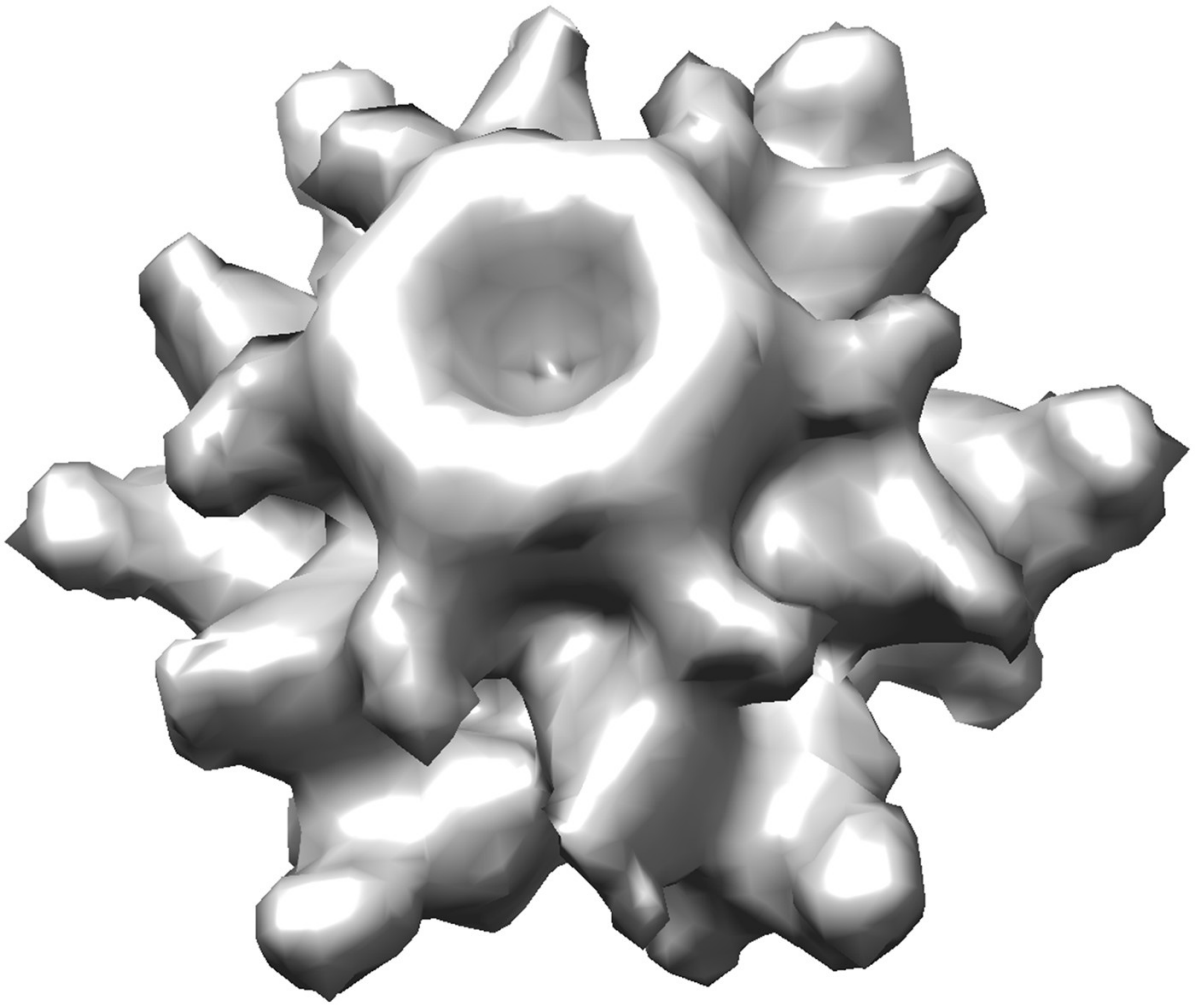
The volumetric rending of cryo-EM structure EM-1706 [67]. Image generated by Chimera [68].

**Fig 9:**
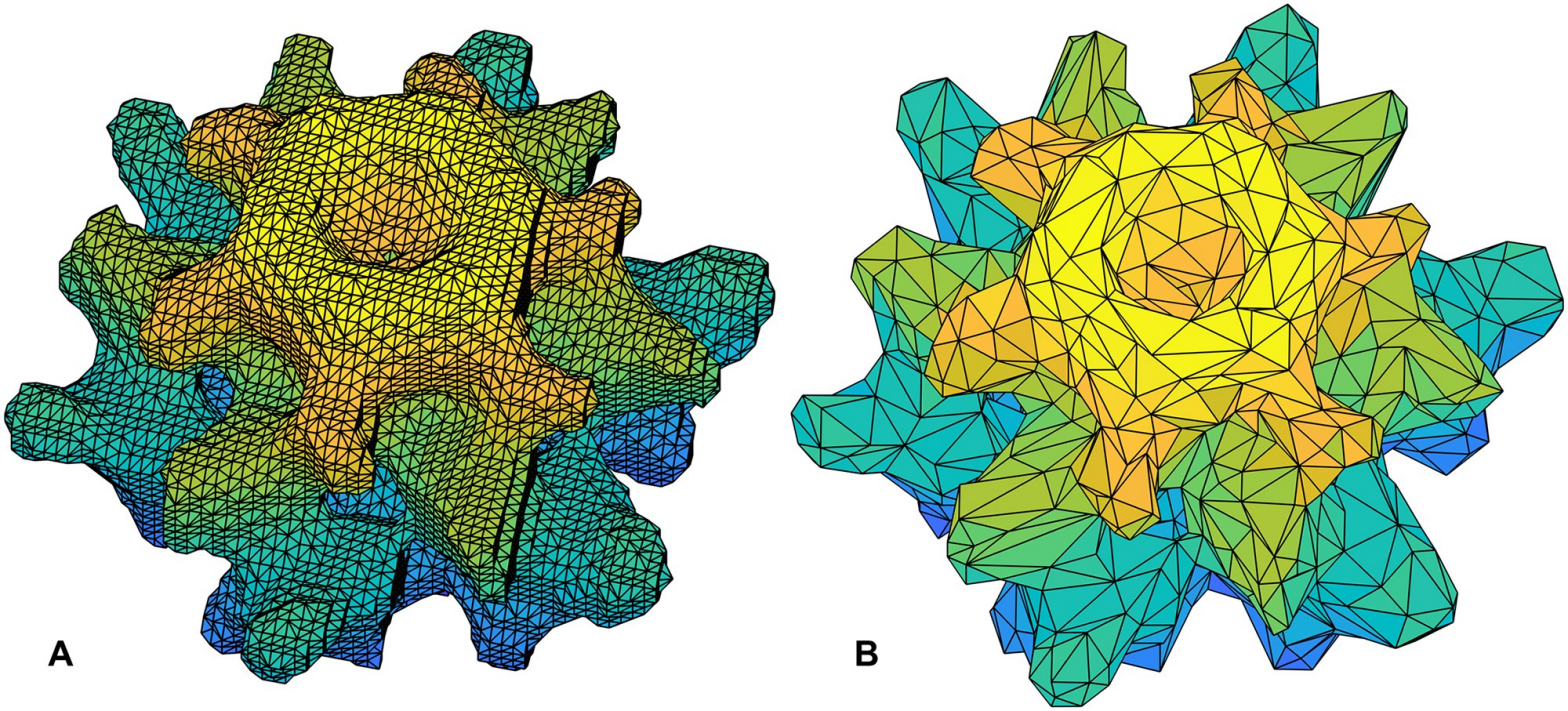
Solid models of the EM structure given in Fig 8 (EM-1706) [67]. (A) A solid model constructed from the original density map using alpha shape. It has 44,561 nodes and 233,564 tetrahedra). (B) A simplified solid model (4,427 nodes, 23,561 tetrahedra).

Alpha shape [19] presents a better solution to this problem as it was designed to capture the shape of a given collection of points. For the EM structure shown in Fig 8 (EM-1706 [67]), Fig 9A shows a solid model obtained by using alpha shape. It uses all the density data points (above a given threshold) as input. The shape captures all the details shown in the original EM map (Fig 8). Additionally, it partitions the structure into a collection of small tetrahedra, which can be readily used in ESM (Fig 1) as finite elements and to compute the stiffness and mass matrices and then normal modes. The algorithm for computing such an alpha shape is highly efficient (nearly linear time to the number of nodes) and the solid model shown in Fig 9A can be obtained in a few seconds on a regular desktop computer.

Moreover, since the number of density data points may be overwhelmingly large for dynamics computations as aforementioned, one often desires to find a simplified representation of the original structure while maintaining the shape. Thankfully, as aforementioned, such problems have been well studied in the computational geometry community and there exist well established algorithms for such a task. For example, take the surface mesh from Fig 9A as input, the MATLAB script given in Fig 6 can be applied to coarse grain the structure while preserving its shape. Fig 9B shows the simplified representation that has only about one-tenth of points on the surface and inside. The new alpha shape again is a collection of many small tetrahedra, a solid model of the original structure.

Given the solid models, ESM can be readily applied to obtain the normal modes. Movies of the first two normal modes (modes 7 and 8) of both the original alpha shape (Fig 9A and the simplified one (Fig 9B) are included in SI (S11, S12, S13 and S14 Videos). Not surprisingly, the two representations, having nearly the identical shape, have similar motion patterns. The computation of the original alpha shape, which contains over 44 thousands nodes, is possible because sparse matrix is used to compute the first few modes.

Major drawbacks of ESM in this context include its dependence on a density cutoff and structure quality (resolution) when generating a surface representation. The solid uniform representation of ESM is limited also in faithfully representing scattered cryo-EM electron density fragments. Lastly, as pointed by one reviewer, the applicability of volumetric models in the field of cryo-EM is much reduced. ESM is no exception.

## Discussion

In this work, we have presented an elastic solid model of macromolecules. The idea should be especially appealing for large structure assemblies, and might be extended to model the structure and dynamics of even larger systems such as organelles or cells, systems at the mesoscopic scale. At that scale, it is probably most appropriate to model a given structure assembly with continuous “protein” or “nucleic acid” materials with certain material properties such as Young’s modulus, or density and elastic tensors that were used in Kinsen’s work on waves in infinite protein crystals [21].

Another appealing feature of ESM is shape-preserving. Shape has long been recognized as a key determinant of dynamics. By employing alpha shape [19], shape is intentionally captured and preserved even during the process of structural coarse-graining. Alpha shape can be used also to compute molecular volume and identify cavities inside a macromolecule [69, 70]. It is foreseeable that ESM can be extended to study the effects of packing density and voids on protein dynamics and function.

Established algorithms exist for simplifying surface/volume while preserving shape in the computational geometry and computer graphics communities. Points on surface are selected strategically instead of uniformly to maintain the surface (or volume) of a structure. Such algorithms developed in these research communities should find useful applications in the field of computational structural biology, for example, in designing non-uniform shape-preserving coarse-graining methods.

ESM by design partitions a given structure into small cells or finite elements and thus naturally inherits and benefits from the much fruit already attained in the field of finite element analysis. For example, ESM should be naturally suited for studying the mechanical response of a system under external force. Experimentally, atomic force microscopy (AFM) has been used to measure the stiffness of viral capsids such as those of bacteriophage *ϕ*29 [40], cowpea chlorotic mottle virus [43], HIV-1 [54], etc. All these studies can be repeated computationally using ESM to better caliber the material properties of the capsids, and in turn, after careful calibration, ESM can be applied to better understand the dynamics of capsids, such as the uncoating of HIV-1 capsids [54].

Another attractive feature of ESM is that it can be applied at ease to cryo-EM density maps and perhaps also some other low-resolution density data.

From its vibrational spectrum (Fig 2), it is seen that ESM modes are significantly different from those of ENM/NMA: they are confined to the low frequency region. High frequency modes in the range up to 3,000 cm^−1^ that were observed in experiments and reproduced in ENM models such as sbNMA [48, 71] are completely missing here. In contrast, one major strength of mass-spring models such as ENM/NMA is that they can accurately capture the vibrations of individual bonds in the high frequency range. Indeed, even small differences between *α* and *β*-rich proteins near the range of amide vibration frequencies were reproduced by ENM models [48], to which ESM might be completely insensitive, as it cares only about the overall shape and material property of a given system.

Computer modeling and simulations at the atomic level have contributed much insight to structural biology in the last few decades. Techniques developed for understanding the mechanistic details of molecular systems during this time, such as molecular dynamics (MD) [72] and normal mode analysis (NMA) [73–75], have been instrumental to the field. Thinking ahead, however, as molecule systems of our interest grow more quickly in size/dimension than what our computation resources can keep up with, some adjustments in our methodology may be necessary. To model and simulate vastly larger systems, we would have to give up atomic modeling in some places, while realizing that it is essential in others. As was allegedly said by Einstein, “Everything should be made as simple as possible, but no simpler.” So the key question is, what should be the most appropriate model for a given system, so that there are enough details for producing meaningful results but not too many to be handled?

If we would ever realize the dream of simulating vastly large systems such as organelles and even cells in a meaningful way, on the modeling side we might have to use hybrid models with both continuous solid components and discrete atomic components. As there are many variants of NMA/ENM, it is foreseeable that better and variants of elastic solid models will be developed. On the analysis side, methods developed for or applied in the microscopic world such as molecular dynamics (MD) and normal mode analysis (NMA) and those developed for the macroscopic world such as finite element analysis (FEA) or computational fluid dynamics (CFD) may need to be integrated somehow to best study systems at the mesoscopic scale, such as cells.

## Supporting information

S1 File The MATLAB script for the ESM model. The file needs to be renamed as ESM.m before use.
(TXT)

S2 File The MATLAB script for coarse-graining a structure represented by a point set. The file needs to be renamed as reduceByLayers.m before use.
(TXT)

S1 Table Detailed results of *α*ESM in interpreting conformational changes using the same benchmark protein dataset and metrics as used in the iMOD work by Chacon and coworkers [13]. (PDF)

S2 Table Detailed results of ANM [5] in interpreting conformational changes using the same benchmark protein dataset and metrics as used in the iMOD work by Chacon and coworkers [13]. (PDF)

S1 Video The first normal mode of ANM of the pig plasma retinol binding protein (183 residues, PDB-id: 1aqb) shown in Table 1. (AVI)

S2 Video The second normal mode of ANM of the pig plasma retinol binding protein (183 residues, PDB-id: 1aqb) shown in Table 1. (AVI)

S3 Video The first normal mode of *α*ESM of the pig plasma retinol binding protein (183 residues, PDB-id: 1aqb) shown in Table 1. (AVI)

S4 Video The second normal mode of *α*ESM of the pig plasma retinol binding protein (183 residues, PDB-id: 1aqb) shown in Table 1. (AVI)

S5 Video The mechanical response of the hexa-hexamer shown in Fig 4 under external force as predicted by ANM. (AVI)

S6 Video The mechanical response of the hexa-hexamer shown in Fig 4 under external force as predicted by *α*ESM. (AVI)

S7 Video The indentation of CCMV capsid by an AFM spherical tip. (MOV)

S8 Video The first *α*ESM mode of HIV-1 capsid. (AVI)

S9 Video The second *α*ESM mode of HIV-1 capsid. (AVI)

S10 Video The 50^*th*^ *α*ESM mode of HIV-1 capsid. (AVI)

S11 Video The first *α*ESM mode of structure EM-1706. (AVI)

S12 Video The second *α*ESM mode of structure EM-1706. (AVI)

S13 Video The first *α*ESM mode of a simplified representation of structure EM-1706. (AVI)

S14 Video The second *α*ESM mode of a simplified representation of structure EM-1706. (AVI)
