# Supplementary material for: Shape-preserving elastic solid models of macromolecules
Source: PLoS Comput Biol. 2020 May 14;16(5):e1007855. doi: 10.1371/journal.pcbi.1007855 (PMC7297265; doi:10.1371/journal.pcbi.1007855)
Supplement: S1 Table — (PDF) [file pcbi.1007855.s003.pdf]

Table S1: Results of αESM in interpreting conformational changes using the same benchmark protein dataset and metrics as used in the iMOD work by Chacon and coworkers. α_1, α_2, α_3: top three best overlap values, δ_3, δ_5, δ_{10}: cumulative overlaps of top 3, 5, 10 modes, $N\alpha_1$: the index of the mode that gives the best overlap, and $N\sigma_{90}$, the number of modes required to cover 90% of the modal variance.

αESM	open to closed								closed to open							
test	α_1	α_2	α_3	δ_3	δ_5	δ_{10}	$N\alpha_1$	$N\sigma_{90\%}$	α_1	α_2	α_3	δ_3	δ_5	δ_{10}	$N\alpha_1$	$N\sigma_{90\%}$
1ex6A	0.75	0.47	0.23	0.92	0.95	0.97	1	11	0.57	0.37	0.36	0.70	0.87	0.92	1	29
4akeA	0.85	0.22	0.21	0.91	0.96	0.98	1	11	0.59	0.35	0.26	0.71	0.75	0.84	1	57
1gggA	0.89	0.22	0.20	0.95	0.96	0.96	1	9	0.74	0.40	0.35	0.83	0.92	0.93	1	31
2laoA	0.95	0.10	0.10	0.96	0.97	0.98	1	7	0.83	0.34	0.10	0.85	0.92	0.93	1	33
1urpA	0.81	0.45	0.20	0.95	0.97	0.98	1	9	0.62	0.52	0.35	0.86	0.93	0.94	1	29
1ramB	0.79	0.47	0.24	0.84	0.98	0.98	1	10	0.66	0.38	0.36	0.80	0.94	0.96	1	12
5at1A	0.65	0.30	0.26	0.74	0.80	0.85	1	163	0.45	0.33	0.31	0.56	0.65	0.79	2	231
1ckmA	0.74	0.52	0.30	0.92	0.97	0.98	1	10	0.58	0.40	0.25	0.67	0.72	0.88	1	69
3dapA	0.64	0.54	0.40	0.86	0.95	0.98	2	11	0.45	0.43	0.37	0.51	0.63	0.89	6	41
1bp5A	0.66	0.66	0.15	0.95	0.96	0.97	1	9	0.76	0.28	0.24	0.83	0.88	0.91	3	51
1jqlA	0.72	0.36	0.26	0.82	0.86	0.89	1	79	0.73	0.37	0.25	0.82	0.86	0.89	1	56
1ompA	0.90	0.27	0.14	0.96	0.96	0.97	1	9	0.80	0.31	0.28	0.91	0.93	0.94	1	23
8adhA	0.61	0.43	0.41	0.87	0.89	0.91	2	65	0.63	0.47	0.35	0.87	0.89	0.91	2	72
9aatA	0.83	0.25	0.22	0.88	0.91	0.94	1	25	0.76	0.31	0.26	0.84	0.88	0.93	1	51
1bncA	0.80	0.39	0.23	0.85	0.94	0.94	1	21	0.67	0.23	0.19	0.70	0.73	0.83	1	86
1dpeA	0.86	0.32	0.19	0.94	0.96	0.97	1	10	0.70	0.37	0.33	0.82	0.89	0.91	1	46
1rkmA	0.85	0.30	0.25	0.89	0.94	0.96	1	13	0.74	0.35	0.29	0.83	0.89	0.90	1	43
1sx4A	0.74	0.40	0.25	0.81	0.90	0.92	1	24	0.61	0.34	0.33	0.73	0.78	0.87	3	53
1i7dA	0.44	0.38	0.37	0.42	0.62	0.75	5	51	0.44	0.37	0.27	0.41	0.53	0.74	7	69
1lfgA	0.56	0.53	0.25	0.61	0.81	0.91	1	36	0.46	0.45	0.39	0.61	0.83	0.93	3	40
1oaoD	0.77	0.44	0.23	0.92	0.94	0.96	2	14	0.82	0.30	0.26	0.88	0.92	0.95	3	16
1ih7A	0.77	0.24	0.22	0.79	0.85	0.92	1	21	0.58	0.37	0.33	0.63	0.72	0.90	3	32
1su4A	0.74	0.31	0.26	0.78	0.82	0.92	1	32	0.60	0.47	0.33	0.63	0.72	0.87	1	64
