# Supplementary material for: Shape-preserving elastic solid models of macromolecules
Source: PLoS Comput Biol. 2020 May 14;16(5):e1007855. doi: 10.1371/journal.pcbi.1007855 (PMC7297265; doi:10.1371/journal.pcbi.1007855)
Supplement: S2 Table — (PDF) [file pcbi.1007855.s004.pdf]

Table S2: Results of ANM in interpreting conformational changes using the same benchmark protein dataset and metrics as used in the iMOD work by Chacon and coworkers.

ANM	open to closed								closed to open							
test	α_1	α_2	α_3	δ_3	δ_5	δ_{10}	$N\alpha_1$	$N\sigma_{90\%}$	α_1	α_2	α_3	δ_3	δ_5	δ_{10}	$N\alpha_1$	$N\sigma_{90\%}$
1ex6A	0.85	0.31	0.18	0.87	0.93	0.94	1	22	0.67	0.37	0.31	0.77	0.83	0.88	1	53
4akeA	0.80	0.35	0.28	0.85	0.95	0.97	1	11	0.54	0.35	0.32	0.61	0.70	0.81	1	468
1gggA	0.88	0.25	0.15	0.92	0.93	0.94	1	31	0.55	0.34	0.29	0.67	0.70	0.82	3	330
2laoA	0.81	0.49	0.15	0.95	0.95	0.97	1	9	0.59	0.37	0.34	0.70	0.72	0.87	3	391
1urpA	0.80	0.52	0.11	0.96	0.97	0.98	2	8	0.69	0.46	0.27	0.87	0.89	0.92	1	85
1ramB	0.59	0.50	0.38	0.86	0.92	0.97	2	12	0.58	0.55	0.37	0.88	0.90	0.96	1	15
5at1A	0.51	0.44	0.36	0.57	0.68	0.82	2	295	0.43	0.32	0.27	0.35	0.48	0.74	7	399
1ckmA	0.90	0.29	0.20	0.94	0.97	0.97	1	10	0.41	0.37	0.35	0.61	0.71	0.88	1	194
3dapA	0.76	0.49	0.28	0.94	0.96	0.97	2	11	0.60	0.33	0.28	0.19	0.30	0.77	8	146
1bp5A	0.79	0.49	0.15	0.94	0.96	0.96	2	10	0.54	0.46	0.25	0.73	0.77	0.84	3	431
1jqlA	0.58	0.49	0.24	0.78	0.79	0.85	1	372	0.60	0.49	0.26	0.78	0.79	0.85	1	275
1ompA	0.77	0.53	0.16	0.94	0.95	0.96	2	11	0.87	0.22	0.21	0.90	0.94	0.94	2	17
8adhA	0.78	0.26	0.19	0.84	0.86	0.87	3	214	0.77	0.20	0.14	0.80	0.81	0.84	3	232
9aatA	0.84	0.24	0.21	0.86	0.88	0.92	1	56	0.82	0.25	0.19	0.84	0.87	0.91	1	82
1bncA	0.86	0.18	0.16	0.89	0.90	0.92	1	96	0.66	0.38	0.20	0.78	0.81	0.87	1	187
1dpeA	0.89	0.31	0.14	0.95	0.97	0.97	1	9	0.71	0.42	0.23	0.75	0.86	0.88	1	167
1rkmA	0.90	0.29	0.14	0.94	0.95	0.97	1	11	0.57	0.37	0.36	0.77	0.82	0.88	2	181
1sx4A	0.84	0.28	0.14	0.84	0.85	0.91	1	83	0.54	0.42	0.31	0.48	0.79	0.84	4	87
1i7dA	0.56	0.34	0.26	0.40	0.69	0.75	5	77	0.51	0.35	0.32	0.35	0.62	0.72	5	165
1lfgA	0.55	0.51	0.27	0.62	0.80	0.86	1	89	0.48	0.46	0.37	0.69	0.86	0.93	1	80
1oaoD	0.62	0.42	0.40	0.85	0.94	0.96	2	14	0.74	0.37	0.30	0.76	0.90	0.95	3	18
1ih7A	0.81	0.32	0.21	0.83	0.85	0.94	1	17	0.61	0.44	0.28	0.68	0.68	0.87	3	36
1su4A	0.73	0.26	0.25	0.79	0.84	0.88	1	112	0.62	0.30	0.24	0.63	0.69	0.78	1	203
